# Long-Term Impact of COVID-19 on Osteoporosis Risk Among Patients Aged ≥50 Years with New-Onset Overweight, Obesity, or Type 2 Diabetes: A Multi-Institutional Retrospective Cohort Study

**DOI:** 10.3390/medicina61081320

**Published:** 2025-07-22

**Authors:** Sheng-You Su, Yi-Fan Sun, Jun-Jun Yeh

**Affiliations:** 1 Clinical Medicine Research Center, Department of Medical Research, Ditmanson Medical Foundation Chia-Yi Christian Hospital, Chiayi City 60002, Taiwan; 15334@cych.org.tw; 2 Department of Family Medicine, Geriatric Medicine, and Clinical Medicine Research Center, Department of Medical Research, Ditmanson Medical Foundation Chia-Yi Christian Hospital, Chiayi City 60002, Taiwan; 07712@cych.org.tw; 3 Department of Thoracic Medicine, Family Medicine, Geriatric Medicine, Medical Research and Medical Education, Ditmanson Medical Foundation Chia-Yi Christian Hospital, Chiayi City 60002, Taiwan

**Keywords:** COVID-19, osteoporosis, bone mineral density, T-score, fragility fractures, obesity, diabetes mellitus, long-term follow-up, retrospective cohort, TriNetX database

## Abstract

*Background and Objectives*: COVID-19 may have long-term adverse effects on bone health, particularly in individuals aged ≥50 years with obesity or diabetes, who are predisposed to impaired bone quality. *Materials and Methods*: This retrospective cohort study used TriNetX data from 141 healthcare organizations across North America and Western Europe. Patients aged ≥50 years with overweight (body mass index 25–30 kg/m^2^), obesity (body mass index ≥ 30 kg/m^2^), or type 2 diabetes (T2DM) and COVID-19 (2019–2024) were propensity score-matched to non-COVID-19 controls. Exclusion criteria included prior overweight, obesity, diabetes, osteoporosis, T-score ≤ −2.5, Z score ≤ −2.0, fractures, pneumonia, tuberculosis, and cancer. Outcomes included new-onset osteoporosis, fragility fractures, and low T-scores (≤−2.5). Cox regression estimated hazard ratios (HRs); sensitivity analyses assessed lag effects (1–4 years). *Results*: Among 327,933 matched pairs, COVID-19 was linked to increased osteoporosis risk at 3 years (HR, 1.039; 95% CI, 1.003–1.077) and 6 years (HR, 1.095; 95% CI, 1.059–1.133). Sensitivity analysis showed rising risk with longer lag times: HRs were 1.212, 1.379, 1.563, and 1.884 at 1 to 4 years, respectively. Subgroup analyses confirmed consistent trends. *Conclusions*: COVID-19 is independently associated with elevated long-term osteoporosis risk in older adults with new-onset overweight, obesity, or T2DM, peaking at 4 years post-infection and persisting through 6 years.

## 1. Introduction

Obesity, diabetes mellitus (DM), and osteoporosis share a complex and interrelated pathophysiological relationship. Individuals with obesity or DM often exhibit normal or even increased bone mineral density (BMD); however, their bone quality is frequently compromised, placing them at elevated risk of fragility fractures [1,2,3]. This paradox may be attributed to increased bone marrow adipose tissue (BMAT), which secretes adipokines and inflammatory mediators that disrupt the bone microenvironment and impair bone metabolism. Moreover, DM has been shown to directly suppress osteoblastic activity while promoting bone marrow adiposity, further reducing bone strength and increasing fracture susceptibility [4].

The coronavirus disease 2019 (COVID-19) pandemic has dramatically impacted global health systems, with emerging evidence indicating that its effects extend beyond the respiratory tract to include bone and mineral metabolism. During the acute phase of infection, severe COVID-19 is associated with a “cytokine storm,” characterized by elevated levels of interleukin-6 (IL-6), tumor necrosis factor-alpha (TNF-α), and other inflammatory markers [5,6]. These cytokines enhance osteoclast differentiation and activity, accelerating bone resorption and contributing to acute bone loss. Additionally, hospitalization-related immobility and the frequent use of corticosteroids in severe COVID-19 cases are known contributors to skeletal deterioration [6,7,8].

In the subacute phase, persistent symptoms, such as post-viral fatigue and limited mobility—hallmarks of long COVID—may perpetuate immobility-induced bone loss [9]. Meanwhile, lingering systemic inflammation and immune dysregulation may continue to suppress bone formation. Nutritional deficiencies, particularly vitamin D insufficiency, which is commonly observed in COVID-19, DM, and obesity, further impair bone remodeling [3,6].

The long-term effects of COVID-19 on bone health are increasingly being recognized. Among patients with pre-existing osteopenia or osteoporosis, COVID-19 may exacerbate disease progression and lead to a more rapid decline in BMD and increased fracture risk. Individuals with DM or obesity—conditions characterized by chronic low-grade inflammation and hormonal/metabolic disturbances—may be particularly susceptible to COVID-19-related skeletal complications [6,10].

Importantly, the interaction between COVID-19, DM, obesity, and osteoporosis represents a synergistic risk pathway. Shared mechanisms, such as chronic inflammation, insulin resistance, physical inactivity, nutritional deficits, and glucocorticoid exposure form a “vicious cycle” that accelerates bone fragility [2,6,11,12]. To date, however, few large-scale studies have quantified the long-term risk of osteoporosis in patients aged ≥50 years with obesity or type 2 diabetes (T2DM) following COVID-19 infection [13].

To address this gap, we conducted a retrospective cohort study using a large, multicenter electronic health record (EHR) database to examine the association between COVID-19 infection and subsequent risk of osteoporosis, low T-score, and fragility fractures in this high-risk population.

## 2. Methods

### 2.1. Study Design

**Figure 1:**
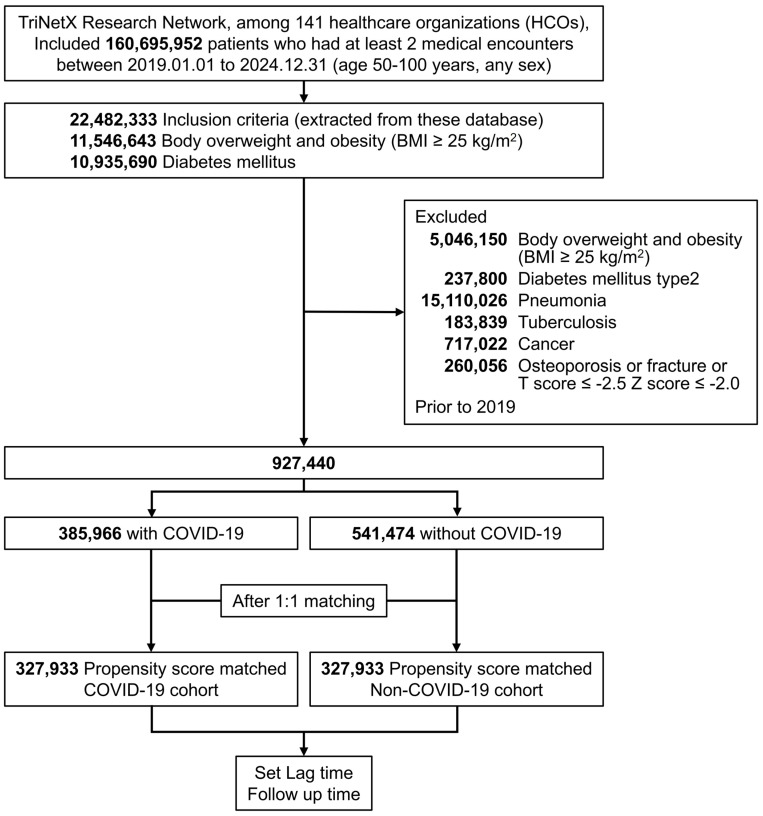
Study flowchart for sample selection. Matching was based on demographics, medical utilization, smoking status, and comorbidities.

This was a retrospective comparative cohort study informed by prior epidemiological frameworks. We compared the risk of newly diagnosed osteoporosis and osteoporosis-related fractures between patients with new-onset overweight, obesity, or T2DM who had COVID-19 and those without COVID-19 (Figure 1).

### 2.2. Data Source

#### Data Source and Study Design

This retrospective cohort study utilized data from the TriNetX Research Network, a global federated health research platform providing secure, web-based access to de-identified electronic medical records. The dataset was extracted for the period spanning 1 January 2025, to 6 June 2025, and included records from 141 healthcare organizations across North America (United States and Canada) and Western Europe (United Kingdom, Germany, France, Spain, and Italy).

TriNetX ensures compliance with ethical standards, including the Health Insurance Portability and Accountability Act (HIPAA) in the United States and the General Data Protection Regulation (GDPR) in Europe. All patient data are de-identified prior to access, and individual informed consent is not required for analyses using the TriNetX platform. Institutional Review Board (IRB) approval was not necessary, as this study involved the analysis of anonymized data only. Nonetheless, the study was approved by the IRB of Chia-Yi Christian Hospital (approval number IRB2025032) and conducted in accordance with the Declaration of Helsinki.

### 2.3. Inclusion and Exclusion Criteria

#### 2.3.1. Inclusion Criteria

Eligible participants were adults aged 50 to 100 years who had a documented diagnosis of new-onset overweight (defined as body mass index [BMI] 25–30 kg/m^2^), obesity (BMI ≥ 30 kg/m^2^), or T2DM, identified using ICD-10-CM codes. Patients with a confirmed diagnosis of COVID-19 between 2020 and 2024 were identified using ICD-10-CM code U07.1 or equivalent codes within the TriNetX platform. The index date was defined as the first recorded diagnosis of COVID-19. To ensure sufficient data for outcome assessment, only patients with at least one follow-up encounter within three months of the index date were included.

#### 2.3.2. Exclusion Criteria

To minimize confounding from pre-existing metabolic or skeletal conditions, patients were excluded if they had any diagnosis of overweight, obesity, or T2DM prior to the index date, as only new-onset cases were considered. Patients with a history of osteoporosis—including a T-score ≤ −2.5 or Z-score ≤ −2.0—or with any osteoporosis-related or pathological fractures were also excluded. Additional exclusions included a history of pneumonia before 2019, tuberculosis, or cancer known to contribute to skeletal fragility or pathological fractures. After applying these criteria, a total of 385,966 patients were included in the final analysis.

### 2.4. Non-COVID-19 Cohort Definition

The non-COVID-19 cohort included individuals without any recorded diagnosis of COVID-19 through 2024 and with at least one healthcare encounter from 2019 to 2024. The index date was the first such encounter. The same exclusion criteria as the COVID-19 cohort were applied. After exclusions, 541,474 eligible non-COVID-19 patients were identified with at least one post-index medical encounter.

### 2.5. Outcomes and Follow-Up

The primary outcome was newly diagnosed osteoporosis (ICD-10) or osteoporosis-related fractures. Follow-up began at the index date and ended at the earliest date of outcome occurrence or the last recorded encounter. Follow-up continued for up to 6 years or until the database cutoff date in December 2024. Additional File 1 (Tables S1–S3) lists the ICD codes and definitions used for exposures and outcomes.

### 2.6. Study Covariates

Baseline covariates included demographics (age, sex, race/ethnicity), comorbidities, healthcare utilization metrics (e.g., emergency and inpatient visits), and select clinical laboratory variables. These variables informed the matching and regression models.

### 2.7. Statistical Analysis

Patient cohorts were propensity-score matched using baseline covariates, including demographics, healthcare utilization, and comorbidities. Logistic regression via scikit-learn was used to estimate propensity scores, and greedy nearest-neighbor matching was performed using a caliper width of 0.1 standard deviations. Matching produced 327,933 pairs.

Kaplan–Meier methods estimated cumulative osteoporosis incidence, with Cox proportional hazards models used for risk comparison. The proportional hazards assumption was tested with the Schoenfeld method, and *p*-values < 0.05 were considered statistically significant. All statistical tests were conducted using the TriNetX Analytics Platform.

### 2.8. Prespecified Outcomes

The primary outcome was new-onset osteoporosis. Time periods were defined as follows: acute (within 3 months post-index), subacute (3–6 months), and chronic (beyond 6 months). Secondary outcomes included low T-score (≤−2.5) and fragility fractures as a positive control. Definitions and codes are provided in Supplementary: Table S3.

### 2.9. Sensitivity Analysis

**Figure 2:**
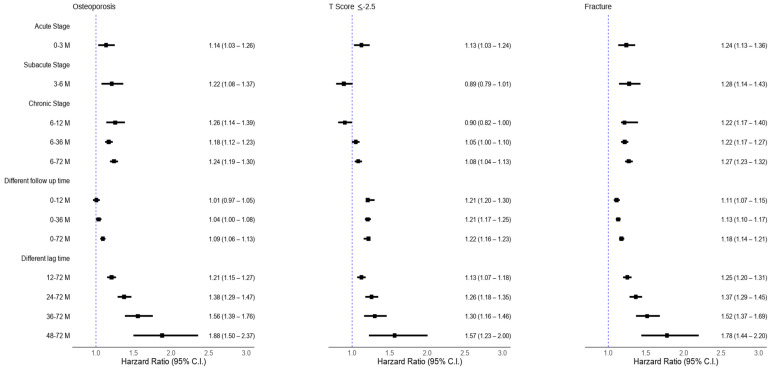
Osteoporosis, low T-scores, and fracture risks between COVID-19 and non-COVID-19 cohorts: Stratified analysis.

To avoid confounding by post-viral syndrome, sensitivity analyses were performed using alternative cutoff times (1, 2, 3, and 4 years) to define osteoporosis, T-score ≤ −2.5, and fractures. Additional tests evaluated the association between COVID-19 and osteoporosis risk with extended follow-up up to 6 years (see Figure 2).

### 2.10. Stratified Analyses

**Figure 3:**
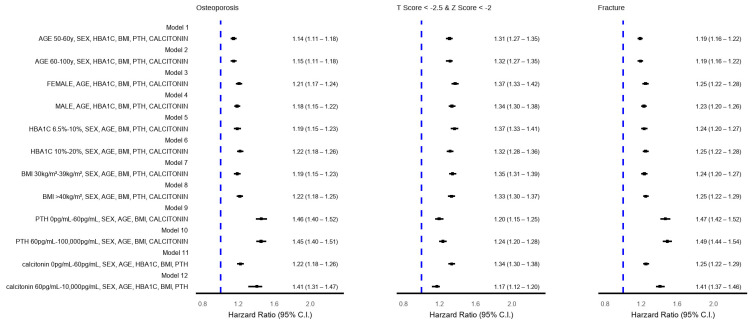
Stratified risk of osteoporosis and related outcomes using 12 statistical models.

Subgroup analyses examined the risk of osteoporosis outcomes by age group (50–60 vs. 60–100 years), sex, BMI (30–39 vs. ≥40), HbA1c (6.5–10 vs. >10), PTH (0–60 vs. >60 pg/mL), and calcitonin (0–60 vs. >60 pg/mL), across 12 propensity score models over 0–6 years. HRs and 95% CIs were calculated for each subgroup (see Figure 3).

## 3. Results

We initially identified 385,966 patients aged ≥50 years and diagnosed with COVID-19 and 541,474 individuals without COVID-19 from 2019 to 2024. After excluding ineligible participants and performing propensity score matching, 327,933 matched pairs were included in the analysis (Figure 1). After matching, demographics, lifestyle factors, comorbidities, medications, and procedures were well balanced between the COVID-19 and non-COVID-19 cohorts (standardized mean difference [SMD] < 0.1), except for HbA1c and calcitonin levels (SMD > 0.1) (Table 1).

Figure 2 presents the risk of osteoporosis over time. Comparing COVID-19 to non-COVID-19 cohorts, the hazard ratios (HRs) with 95% confidence intervals (CIs) were as follows: 1.009 (95% CI: 0.966–1.054) at 1 year, 1.039 (95% CI: 1.003–1.077) at 3 years, and 1.095 (95% CI: 1.059–1.133) at 6 years. Differences at 3 and 6 years were statistically significant (*p* < 0.05), and HRs increased with a longer follow-up.

Sensitivity analyses using 1-, 2-, 3-, and 4-year post-index cutoffs showed consistent HRs of 1.212 (95% CI: 1.154–1.273), 1.379 (95% CI: 1.290–1.474), 1.563 (95% CI: 1.388–1.759), and 1.884 (95% CI: 1.501–2.366), respectively, supporting the association between COVID-19 and new-onset osteoporosis.

Secondary outcomes also demonstrated positive associations: patients with COVID-19 had lower T-scores (≤−2.5) and a higher incidence of fractures compared to the non-COVID-19 cohort (Figure 2).

Figure 3 summarizes HRs from 12 stratified PSM models across subgroups and follow-up periods. Elevated HRs were observed across age (50–60 vs. 60–100 years), sex, HbA1c, BMI, PTH, and calcitonin levels, indicating consistent increased risk of osteoporosis, low T-score, and fractures in the COVID-19 cohort. All subgroup comparisons were statistically significant (*p* < 0.005).

**Figure 4:**
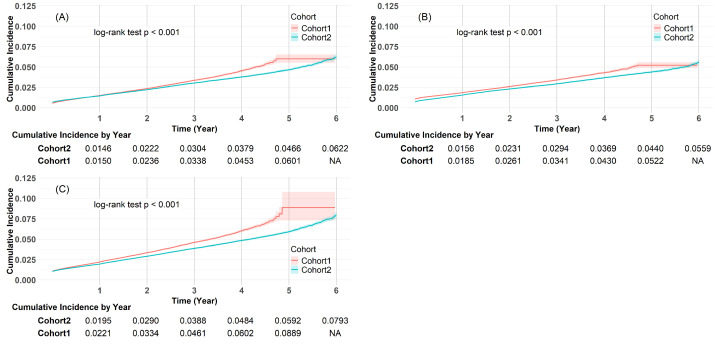
Longitudinal analysis of the cumulative incidence of osteoporosis in COVID-19 (Cohort 1) vs. non-COVID-19 (Cohort 2) cohorts: (**A**) osteoporosis; (**B**) T-score ≤ −2.5; (**C**) fractures.

Kaplan–Meier analysis further demonstrated significantly higher cumulative incidence of osteoporosis (log-rank *p* < 0.001; Figure 4A), low T-score (Figure 4B), and fractures (Figure 4C) in the COVID-19 cohort.

## 4. Discussion

### 4.1. Key Findings

In our cohort study, COVID-19 patients were significantly more likely to be diagnosed with osteoporosis within the first 3 months or during extended follow-up at multiple intervals, namely 6, 12, 24, 36, 48, and 60 months after infection, compared to propensity score-matched patients without COVID-19. Importantly, the elevated hazard ratios (HRs) persisted regardless of whether the analysis was initiated at the index date or 3 months, 1 year, 2 years, 3 years, or even 4 years after the COVID-19 diagnosis. This robust finding, which minimizes the risk of reverse causality, underscores the long-term impact of COVID-19 on bone health.

### 4.2. Comparison with the Existing Literature

A Hong Kong study with a median follow-up of 11 months similarly demonstrated that COVID-19 patients had a higher risk of major osteoporotic fractures (HR 1.22, 95% CI [1.15–1.31]) [14]. Both the acute phase (within 30 days) and post-acute phase (beyond 30 days) were associated with significantly higher risks of fractures and falls. In parallel, a UK study reported a significant reduction in T-score (mean change of −0.23) and increased risk of osteoporosis (OR 1.49, 95% CI [1.40–1.59]) post-COVID-19, consistent with our findings [12].

### 4.3. Clinical Implications

To address ascertainment bias, we excluded osteoporosis diagnoses made within 1–4 years post-COVID-19. Despite these lag times, the risk remained elevated in the COVID-19 group, with the highest HR observed at a 4-year lag. The risk increased progressively over longer follow-up periods.

Subgroup analyses revealed that even individuals aged 50–60 years and those with high HbA1c (>10), BMI (30–39, >40), PTH (>60), and calcitonin (>60) had significantly increased HRs. COVID-19, obesity, and DM may share common inflammatory and metabolic pathways contributing to compromised bone health [1,3,10,15]. Weight loss, immobilization, and prolonged bed rest could exacerbate bone loss, while uncontrolled DM impairs bone quality and healing [11,12,14,15,16,17,18,19].

Our results suggest that COVID-19 is associated with acute, subacute, and chronic phases of osteoporosis risk. Clinical strategies should incorporate individualized assessments and advanced osteoporosis therapies for high-risk individuals.

### 4.4. Strengths

This study demonstrates that COVID-19 is associated with a long-term rise in osteoporosis, low BMD, and fracture risks among patients with new-onset overweight, obesity, or T2DM. These risks increased over time, peaking at 4 years post-infection, and were consistent across sensitivity and subgroup analyses.

The extended follow-up (6 to 60 months) allowed the assessment of evolving risk and helped distinguish incidence from pre-existing osteoporosis. Propensity score matching reduced confounding, and the large sample size supported generalizability.

### 4.5. Limitations

This study’s retrospective nature and reliance on EHR data carry risks of selection bias, coding inconsistencies, and limited granularity. Our cohort may not represent underserved populations. Important variables (e.g., corticosteroid use, lifestyle, and vitamin D status) were unavailable.

Future work should incorporate broader populations, clinical variables, and long-term surveillance to strengthen causal inferences.

## 5. Conclusions

This large, multicenter retrospective cohort study demonstrates that COVID-19 is significantly associated with the long-term risk of osteoporosis, low T-scores, and fragility fractures in patients aged ≥50 years with new-onset overweight, obesity or T2DM. These risks were higher in the COVID-19 cohort than in the non-COVID-19 cohort over time, peaking at 4 years post-infection and remained consistent across sensitivity and subgroup analyses. These findings highlight the need for vigilant bone health monitoring and early intervention strategies in this vulnerable population. As the pandemic’s long-term sequelae continue to unfold, healthcare systems must prioritize integrated care pathways that address not only cardiopulmonary complications but also musculoskeletal health in post-COVID-19 survivors.

## Figures and Tables

**Table 1:** Comparison of baseline characteristics between COVID-19 and non-COVID patients aged ≥50 years before and after matching.

	Cohort Before Matching, No (%)			Cohort After Matching, No (%)		
Characteristics	COVID-19 (*n* = 385, 966)	Non-COVID (*n* = 541, 474)	SMD	COVID-19 (*n* = 327, 933)	Non-COVID (*n* = 327, 933)	SMD
Age						
Age 50–65	20,651 (5)	38,540 (7)	0.07	16,747 (5)	15,965 (5)	0.01
Age 65–85	209,651 (54)	299,712 (55)	0.02	178,842 (55)	179,264 (55)	0.00
Age 85–100	155,684 (41)	203,222 (38)	0.06	132,344 (40)	132,704 (40)	0.00
Sex						
Female	217,901 (56)	301,072 (56)	0.02	184,563 (56)	184,109 (56)	0.00
Male	161,826 (42)	230,671 (43)	0.01	138,204 (42)	138,857 (42)	0.00
Unknown	7720 (2)	6830 (1)	0.01	6539 (2)	6560 (2)	0.00
Race						
White	238,520 (62)	317,132 (59)	0.07	201,171 (61)	201,305 (61)	0.00
Black	68,843 (18)	97,046 (18)	0.00	58,527 (18)	58,262 (18)	0.00
Asian	21,463 (6)	30,983 (6)	0.01	19,089 (6)	19,398 (6)	0.00
Other	21,816 (6)	35,720 (7)	0.04	18,836 (6)	19,375 (6)	0.01
Unknown	31,352 (8)	55,319 (10)	0.07	29,927 (9)	29,514 (9)	0.01
Ethnicity						
Hispanic or Latino	46,352 (12)	66,500 (12)	0.01	38,984 (12)	40,295 (12)	0.01
Not Hispanic or Latino	300,903 (78)	399,148 (74)	0.10	255,300 (78)	256,018 (78)	0.00
Unknown	38,644 (10)	75,826 (14)	0.12	33,649 (10)	31,620 (10)	0.02
Basic laboratory data	Mean (SD)	Mean (SD)		Mean (SD)	Mean (SD)	
Blood pressure, systolic, mean (SD), mmHg	128 (19)	128 (19)	0.02	128 (19)	128 (19)	0.02
Blood pressure, diastolic, mean (SD), mmHg	76 (12)	77 (12)	0.03	77 (12)	77 (12)	0.01
Calcium, mean (SD), mg/dL	9 (1)	9 (1)	0.08	9 (1)	9 (1)	0.04
Glomerular filtration rate, mean (SD), mL/min/1.73 m^2^	79 (26)	79 (25)	0.03	79 (26)	79 (25)	0.02
Hemoglobin, mean (SD), g/dL	13 (2)	13 (2)	0.07	13 (2)	13 (2)	0.03
Albumin, mean (SD), g/dL	4 (0)	4 (0)	0.11	4 (0)	4 (0)	0.06
Cholesterol in HDL, mean (SD), mg/dL	52 (16)	53 (16)	0.03	52 (16)	53 (16)	0.03
Cholesterol, mean (SD), mg/dL	180 (45)	182 (45)	0.05	180 (46)	182 (45)	0.04
Triglyceride, mean (SD), mg/dL	135 (93)	134 (91)	0.01	136 (93)	134 (92)	0.02
Cholesterol in LDL, mean (SD), mg/dL	104 (36)	107 (36)	0.07	104 (36)	106 (36)	0.05
Thyrotropin, mean (SD), U/L	4 (31)	4 (30)	0.00	4 (32)	4 (30)	0.01
Hemoglobin A1c, mean (SD), %	6 (1)	6 (1)	0.05	6 (1)	6 (1)	0.13
Phosphate, mean (SD), mg/dL	4 (1)	4 (1)	0.04	4 (1)	4 (1)	0.02
Parathyrin. intact, mean (SD), pg/mL	111 (189)	101 (158)	0.06	109 (196)	101 (158)	0.05
Left ventricle ejection fraction, mean (SD), %	58 (11)	59 (11)	0.02	58 (11)	58 (11)	0.01
Body mass index percentile, mean (SD), %	73 (30)	79 (27)	0.20	74 (30)	73 (30)	0.06
Albumin/creatinine ratio, mean (SD), mg/g	246 (6337)	99 (454)	0.03	276 (6907)	97 (465)	0.04
Cortisol, mean (SD), ug/dL	11 (7)	11 (7)	0.08	11 (7)	11 (7)	0.01
Calcitonin, mean (SD), pg/mL	1400 (6408)	138 (480)	0.28	172 (417)	97 (180)	0.23
Comorbidities and condition						
Endocrine, nutritional, and metabolic diseases	152,109 (39)	117,614 (22)	0.39	99,488 (30)	97,788 (30)	0.01
Hyperlipidemia, unspecified	51,422 (13)	42,619 (8)	0.18	36,932 (11)	36,096 (11)	0.01
Body mass index	44,847 (12)	31,356 (6)	0.21	24,912 (8)	24,728 (8)	0.00
Other long-term drug therapies	34,679 (9)	28,356 (5)	0.15	24,448 (7)	24,187 (7)	0.00
Other symptoms and signs involving the digestive system	13,771 (4)	11,336 (2)	0.09	10,240 (3)	8875 (3)	0.02
Nicotine dependence, unspecified	8013 (2)	7067 (1)	0.06	5597 (2)	5555 (2)	0.00
Dyspnea, unspecified	7369 (2)	5418 (1)	0.08	5218 (2)	4382 (1)	0.02
Gout	5387 (1)	4435 (1)	0.06	3771 (1)	3686 (1)	0.00
Alcohol-related disorders	5197 (1)	3926 (1)	0.06	3413 (1)	3325 (1)	0.00
Systemic connective tissue disease	4416 (1)	4128 (1)	0.04	3305 (1)	3243 (1)	0.00
Long-term use of steroids	4245 (1)	3001 (1)	0.06	2691 (1)	2654 (1)	0.00
Exercise counseling	3852 (1)	2888 (1)	0.05	2436 (1)	2433 (1)	0.00
Alcohol abuse	2945 (1)	2148 (1)	0.05	1870 (1)	1808 (1)	0.00
Long-term use of non-steroidal anti-inflammatories	2556 (1)	2020 (0)	0.04	1747 (1)	1734 (1)	0.00
Long-term use of systemic steroids	2421 (1)	1741 (0)	0.04	1563 (0)	1526 (0)	0.00
Long-term use of inhaled steroids	2180 (1)	1486 (0)	0.04	1329 (0)	1326 (0)	0.00
Influenza due to influenza virus	1491 (0)	1360 (0)	0.02	1073 (0)	1016 (0)	0.00
Body mass index of 19.9 or less, adult ^a^	1109 (0)	733 (0)	0.03	698 (0)	673 (0)	0.00
Overweight and obesity	39,870 (10)	589 (0)	0.47	599 (0)	589 (0)	0.00
Pressure ulcer	1140 (0)	480 (0)	0.05	465 (0)	457 (0)	0.00
Abnormal findings on diagnostic imaging of limbs	368 (0)	342 (0)	0.01	272 (0)	280 (0)	0.00
Abnormal results of pulmonary function studies	331 (0)	289 (0)	0.01	240 (0)	231 (0)	0.00
Morbid (severe) obesity due to excess calories	6089 (2)	191 (0)	0.17	204 (0)	179 (0)	0.00
Pneumonia, unspecified organism	4125 (1)	74 (0)	0.14	79 (0)	74 (0)	0.00
Procedure						
Immunology procedures	31,913 (8)	29,034 (5)	0.12	23,607 (7)	23,439 (7)	0.00
Vaccination for COVID-19 ^b^	17,296 (4)	11,201 (2)	0.14	11,030 (3)	10,669 (3)	0.01
Immunization for COVID-19 ^b^	12,484 (3)	7709 (1)	0.12	7667 (2)	7344 (2)	0.01
Dual-energy X-ray absorptiometry (DXA), bone density study	6704 (2)	5686 (1)	0.06	5005 (2)	4961 (2)	0.00
Smoking and tobacco use cessation counseling visit	997 (0)	867 (0)	0.02	699 (0)	689 (0)	0.00
Smoking and tobacco use cessation counseling visit; intermediate	861 (0)	752 (0)	0.02	607 (0)	598 (0)	0.00
Dual-energy X-ray absorptiometry (DXA), appendicular skeleton	275 (0)	242 (0)	0.01	216 (0)	200 (0)	0.00
Dual-energy X-ray absorptiometry (DXA), axial skeleton	254 (0)	259 (0)	0.01	194 (0)	173 (0)	0.00
Admission procedure	110 (0)	244 (0)	0.01	93 (0)	94 (0)	0.00
Admission to establishment	110 (0)	244 (0)	0.01	93 (0)	94 (0)	0.00
Hospital admission	52 (0)	170 (0)	0.01	48 (0)	57 (0)	0.00
Emergency room admission	50 (0)	143 (0)	0.01	46 (0)	56 (0)	0.00
Admission to department	58 (0)	81 (0)	0.00	45 (0)	38 (0)	0.00
Admission to:						
Intensive care unit	10 (0)	20 (0)	0.00	10 (0)	10 (0)	0.00
Neurology department	10 (0)	10 (0)	0.00	10 (0)	0 (0)	0.01
Pulmonary medicine department	14 (0)	10 (0)	0.00	10 (0)	10 (0)	0.00
Intensive care unit	0 (0)	0 (0)	0.00	0 (0)	0 (0)	0.00
Medications						
Central nervous system drugs	166,002 (43)	177,022 (33)	0.21	130,697 (40)	129,547 (40)	0.01
SARS-CoV-2 (COVID-19) vaccine	101,880 (26)	103,641 (19)	0.17	80,137 (24)	78,305 (24)	0.01
Antibacterials for systemic use	102,532 (27)	107,062 (20)	0.16	79,341 (24)	79,331 (24)	0.00
Drug for obstructive lung disease	93,262 (24)	95,068 (18)	0.16	71,515 (22)	71,408 (22)	0.00
Cardiac therapy	84,989 (22)	82,546 (15)	0.17	64,018 (20)	63,490 (19)	0.00
Lipid-modifying agent	66,783 (17)	74,096 (14)	0.10	54,228 (17)	52,754 (16)	0.01
Sedatives or hypnotics	49,608 (13)	47,233 (9)	0.13	37,082 (11)	36,586 (11)	0.00
Antithrombotic agents	48,929 (13)	46,295 (9)	0.13	36,134 (11)	35,162 (11)	0.01
Benzodiazepine derivatives	42,010 (11)	39,634 (7)	0.12	31,157 (10)	30,759 (9)	0.00
Drugs used in diabetes	28,240 (7)	35,641 (7)	0.03	23,902 (7)	22,494 (7)	0.02
Non-steroidal anti-inflammatory drugs	25,519 (7)	28,273 (5)	0.06	19,728 (6)	19,501 (6)	0.00
Antihypertensives	14,446 (4)	12,877 (2)	0.08	10,140 (3)	10,054 (3)	0.00
Endocrine therapy	11,559 (3)	13,759 (3)	0.03	9225 (3)	8987 (3)	0.00
Immunosuppressants	8835 (2)	9376 (2)	0.04	7080 (2)	6974 (2)	0.00
Colchicine	2144 (1)	2200 (0)	0.02	1673 (1)	1647 (1)	0.00
Anti-obesity preparations	1796 (0)	1807 (0)	0.02	1344 (0)	1293 (0)	0.00
Antimycobacterial	857 (0)	959 (0)	0.01	653 (0)	655 (0)	0.00

Abbreviations: ^a^ Body mass index, calculated as weight in kilograms divided by height in meters squared; ^b^ COVID-19, Coronavirus disease 2019.

## Data Availability

The data presented in this study are available on request from the corresponding author. The data are not publicly available due to institutional policies and patient privacy regulations.

## Supplementary Materials

The following supporting information can be downloaded at https://www.mdpi.com/article/10.3390/medicina61081320/s1, including: Table S1: Diagnosis code definitions. Table S2: Propensity score matching covariates. Table S3: Osteoporosis, T-score, and fracture ICD-10 definitions.

## References

[1] Guimarães G.C., Coelho J.B.C., Silva J.G.O., de Sant’Ana A.C.C., de Sá C.A.C., Moreno J.M., Reis L.M., de Oliveira Guimarães C.S. (2024). Obesity, diabetes and risk of bone fragility: How BMAT behavior is affected by metabolic disturbances and its influence on bone health. Osteoporos. Int..

[2] Martiniakova M., Biro R., Penzes N., Sarocka A., Kovacova V., Mondockova V., Omelka R. (2024). Links among Obesity, Type 2 Diabetes Mellitus, and Osteoporosis: Bone as a Target. Int. J. Mol. Sci..

[3] Anghel L., Manole C., Nechita A., Tatu A.L., Ștefănescu B.I., Nechita L., Bușilă C., Zainea P., Baroiu L., Mușat C.L. (2023). Calcium, Phosphorus and Magnesium Abnormalities Associated with COVID-19 Infection, and Beyond. Biomedicines.

[4] Lopez N., Cohen S.M., Emanuele M. (2023). Type 2 Diabetes and Bone Disease. Clin. Rev. Bone Miner. Metab..

[5] Haudenschild A.K., Christiansen B.A., Orr S., Ball E.E., Weiss C.M., Liu H., Fyhrie D.P., Yik J.H.N., Coffey L.L., Haudenschild D.R. (2023). Acute bone loss following SARS-CoV-2 infection in mice. J. Orthop. Res..

[6] Alghamdi F., Mokbel K., Meertens R., Obotiba A.D., Alharbi M., Knapp K.M., Strain W.D. (2024). Bone Mineral Density, Bone Biomarkers, and Joints in Acute, Post, and Long COVID-19: A Systematic Review. Viruses.

[7] Huang C., Huang L., Wang Y., Li X., Ren L., Gu X., Kang L., Guo L., Liu M., Zhou X. (2021). 6-month consequences of COVID-19 in patients discharged from hospital: A cohort study. Lancet.

[8] Tahtabasi M., Kilicaslan N., Akin Y., Karaman E., Gezer M., Icen Y.K., Sahiner F. (2021). The Prognostic Value of Vertebral Bone Density on Chest CT in Hospitalized COVID-19 Patients. J. Clin. Densitom..

[9] Elmedany S.H., Badr O.I., Abu-Zaid M.H., Tabra S.A.A. (2022). Bone mineral density changes in osteoporotic and osteopenic patients after COVID-19 infection. Egypt. Rheumatol. Rehabil..

[10] Soeroto A.Y., Soetedjo N.N., Purwiga A., Santoso P., Kulsum I.D., Suryadinata H., Ferdian F. (2020). Effect of increased BMI and obesity on the outcome of COVID-19 adult patients: A systematic review and meta-analysis. Diabetes Metab. Syndr..

[11] Harris A., Creecy A., Awosanya O.D., McCune T., Ozanne M.V., Toepp A.J., Kacena M.A., Qiao X. (2024). SARS-CoV-2 and its Multifaceted Impact on Bone Health: Mechanisms and Clinical Evidence. Curr. Osteoporos. Rep..

[12] Amin H., Khan M.A., Bukhari M. (2024). Investigating the impact of COVID-19 lockdowns on fragility fracture risk and bone mineral density in a large observational cohort: A cross-sectional study. Rheumatol. Adv. Pract..

[13] Tang J. (2022). COVID-19 Pandemic and Osteoporosis in Elderly Patients. Aging Dis.

[14] Lui D.T.W., Xiong X., Cheung C.-L., Lai F.T.T., Li X., Wan E.Y.F., Chui C.S.L., Chan E.W.Y., Cheng F.W.T., Chung M.S.H. (2024). Risks of incident major osteoporotic fractures following SARS-CoV-2 infection among older individuals: A population-based cohort study in Hong Kong. J. Bone Miner. Res..

[15] Galliera E., Massaccesi L., Mangiavini L., De Vecchi E., Villa F., Corsi Romanelli M.M., Peretti G. (2024). Effects of COVID-19 on bone fragility: A new perspective from osteoimmunological biomarkers. Front. Immunol..

[16] Sapra L., Saini C., Garg B., Gupta R., Verma B., Mishra P.K., Srivastava R.K. (2022). Long-term implications of COVID-19 on bone health: Pathophysiology and therapeutics. Inflamm. Res..

[17] Gittoes N.J., Criseno S., Appelman-Dijkstra N.M., Bollerslev J., Canalis E., Rejnmark L., Hassan-Smith Z. (2020). ENDOCRINOLOGY IN THE TIME OF COVID-19: Management of calcium metabolic disorders and osteoporosis. Eur. J. Endocrinol..

[18] Berktaş B.M., Gökçek A., Hoca N.T., Koyuncu A. (2022). COVID-19 illness and treatment decrease bone mineral density of surviving hospitalized patients. Eur. Rev. Med. Pharmacol. Sci..

[19] Wang Z., Li Z., Shen Y., Qian S., Tang M., He J., Lu H., Zhang N. (2024). Long-term effects of COVID-19 infection on bone mineral density. J. Glob. Health.

