# Supplementary material for: Long-Term Impact of COVID-19 on Osteoporosis Risk Among Patients Aged ≥50 Years with New-Onset Overweight, Obesity, or Type 2 Diabetes: A Multi-Institutional Retrospective Cohort Study
Source: Medicina (Kaunas). 2025 Jul 22;61(8):1320. doi: 10.3390/medicina61081320 (PMC12387685; doi:10.3390/medicina61081320)
Supplement: Supplementary file 1 [file medicina-61-01320-s001.zip › medicina-3721198-supplementary.pdf]

Additional file 1:

1. **Table S1. Diagnostic, procedural, and laboratory codes used in the definition of the cohorts ,inclusion and exclusion criteria.**
2. **Table S2. Demographic, diagnostic, laboratory, and medication codes used in the definition of covariates.**
3. **Table S3. Diagnostic codes used in the definition of outcomes.**

TABLE S1

COVID-19 Cohort

Study Population for covid-19 cohort			
COVID19	diagnosis	UMLS:ICD10CM:U07.1	COVID-19
		UMLS:ICD10CM:J12.82	Pneumonia due to coronavirus disease 2019
	or		
	laboratory	UMLS:LNC:94309-2	SARS-CoV-2 (COVID-19) RNA [Presence] in Specimen by NAA with probe detection
	laboratory	TNX:9088	SARS coronavirus 2 and related RNA [Presence]
		UMLS:LNC:94309-2	SARS-CoV-2 (COVID-19) RNA [Presence] in Specimen by NAA with probe detection (labResult: Positive)
		UMLS:LNC:94316-7	SARS-CoV-2 (COVID-19) N gene [Presence] in Specimen by NAA with probe detection (labResult: Positive)
		UMLS:LNC:94500-6	SARS-CoV-2 (COVID-19) RNA [Presence] in Respiratory system specimen by NAA with probe detection (labResult: Positive)
		UMLS:LNC:94533-7	SARS-CoV-2 (COVID-19) N gene [Presence] in Respiratory system specimen by NAA with probe detection (labResult: Positive)
		UMLS:LNC:94559-2	SARS-CoV-2 (COVID-19) ORF1ab region [Presence] in Respiratory system specimen by NAA with probe detection (labResult: Positive)
		UMLS:LNC:94558-4	SARS-CoV-2 (COVID-19) Ag [Presence] in Respiratory system specimen by Rapid immunoassay (labResult: Positive)
		UMLS:LNC:94534-5	ARS-CoV-2 (COVID-19) RdRp gene [Presence] in Respiratory system specimen by NAA with probe detection (labResult: Positive)
		UMLS:LNC:94565-9	ARS-CoV-2 (COVID-19) RNA [Presence] in Nasopharynx by NAA with non-probe detection (labResult: Positive)
		UMLS:LNC:94639-2	SARS-CoV-2 (COVID-19) ORF1ab region [Presence] in Specimen by NAA with probe detection (labResult: Positive)
		UMLS:LNC:94759-8	SARS-CoV-2 (COVID-19) RNA [Presence] in Nasopharynx by NAA with probe detection (labResult: Positive)
		UMLS:LNC:94763-0	SARS-CoV-2 (COVID-19) [Presence] in Specimen by Organism specific culture (labResult: Positive)
		MLS:LNC:94760-6	SARS-CoV-2 (COVID-19) N gene [Presence] in Nasopharynx by NAA with probe detection (labResult: Positive)

		UMLS:LNC:94758-0	SARS-related coronavirus E gene [Presence] in Respiratory system specimen by NAA with probe detection (labResult: Positive)
		UMLS:LNC:95406-5	SARS-CoV-2 (COVID-19) RNA [Presence] in Nose by NAA with probe detection (labResult: Positive)
		UMLS:LNC:95409-9	SARS-CoV-2 (COVID-19) N gene [Presence] in Nose by NAA with probe detection (labResult: Positive)
		UMLS:LNC:95424-8	SARS-CoV-2 (COVID-19) RNA [Presence] in Respiratory system specimen by Sequencing (labResult: Positive)
		UMLS:LNC:95608-6	SARS-CoV-2 (COVID-19) RNA [Presence] in Respiratory system specimen by NAA with non-probe detection (labResult: Positive)
		UMLS:LNC:96119-3	SARS-CoV-2 (COVID-19) Ag [Presence] in Upper respiratory specimen by Immunoassay (labResult: Positive)
		UMLS:LNC:96123-5	SARS-CoV-2 (COVID-19) RdRp gene [Presence] in Upper respiratory specimen by NAA with probe detection (labResult: Positive)
		UMLS:LNC:96763-8	SARS-CoV-2 (COVID-19) E gene [Presence] in Respiratory system specimen by NAA with probe detection (labResult: Positive)
		UMLS:LNC:97097-0	SARS-CoV-2 (COVID-19) Ag [Presence] in Upper respiratory specimen by Rapid immunoassay (labResult: Positive)

Definition of other inclusion and exclusion criteria

OBESITY AND OVERWEIGHT	diagnosis	UMLS:ICD10CM:E66	Overweight and obesity
DIABETES TYPE 2	diagnosis	UMLS:ICD10CM: E11	Diabetes mellitus TYPE 2
OSTEOPOROSIS/FRACTURE	diagnosis	UMLS:ICD10CM:M81	Osteoporosis without current pathological fracture
	diagnosis	UMLS:ICD10CM:M81.0	Age-related osteoporosis without current pathological fracture
	procedure	UMLS:SNOMED:391027005	Osteoporosis - falls prevention
	diagnosis	UMLS:ICD10:s32	Fracture of lumbar spine and pelvis
	diagnosis	UMLS:ICD10:s42	Fracture of shoulder and upper arm
	diagnosis	UMLS:ICD10:s72	Fracture of femur
	diagnosis	UMLS:ICD10:s82	Fracture of lower leg
PNEUMONIA	diagnosis	UMLS:ICD10CM:J18	Pneumonia, unspecified organism
TUBERCULOSIS	diagnosis	UMLS:ICD10CM:A15-A19	Tuberculosis
CANCER	diagnosis	UMLS:ICD10CM:C80.1	Malignant (primary) neoplasm, unspecified

TABLE S2

TNX curated code in table 1

**Demographics**	
2054-5	Black or African American
M	Male
2106-3	White
1002-5	American Indian or Alaska Native
UNK	Unknown Race
2076-8	Native Hawaiian or Other Pacific Islander
UN	Unknown Gender/Ethnicity
2135-2	Hispanic or Latino
2186-5	Not Hispanic or Latino
2131-1	Other Race
2028-9	Asian
E66	Overweight and obesity
Z68-Z68	Body mass index [BMI] (Z68)
E66.01	Morbid (severe) obesity due to excess calories
F17.200	Nicotine dependence, unspecified, uncomplicated
F10	Alcohol related disorders
F10.1	Alcohol abuse
M10	Gout
R93.6	Abnormal findings on diagnostic imaging of limbs
M30-M36	Systemic connective tissue disorders
E78.5	Hyperlipidemia, unspecified
Z79.51	Long term (current) use of inhaled steroids
Z79.52	Long term (current) use of systemic steroids
Z79.5	Long term (current) use of steroids
Z79.899	Other long term (current) drug therapy
Z79.1	Long term (current) use of non-steroidal anti-inflammatories (NSAID)
Z71.82	Exercise counseling
Z68.1	Body mass index [BMI] 19.9 or less, adult
J18	Pneumonia, unspecified organism
R06.00	Dyspnea, unspecified
R94.2	Abnormal results of pulmonary function studies
R19	Other symptoms and signs involving the digestive system and abdomen
E00-E89	Endocrine, nutritional and metabolic diseases

L89	Pressure ulcer
J10.1	Influenza due to other identified influenza virus with other respiratory manifestations
1018513	Smoking and tobacco use cessation counseling visit
99406	Smoking and tobacco use cessation counseling visit; intermediate, greater than 3 minutes up to 10 minutes
305056002	Admission procedure
305335007	Admission to establishment
305349003	Admission to department
305351004	Admission to intensive care unit
305379005	Admission to neurology department
305352006	Admission to adult intensive care unit
305360007	Admission to pulmonary medicine department
32485007	Hospital admission
50849002	Emergency room admission
77081	Dual-energy X-ray absorptiometry (DXA), bone density study, 1 or more sites; appendicular skeleton (peripheral) (eg, radius, wrist, heel)
1014948	Dual-energy X-ray absorptiometry (DXA), bone density study, 1 or more sites
77085	Dual-energy X-ray absorptiometry (DXA), bone density study, 1 or more sites; axial skeleton (eg, hips, pelvis, spine), including vertebral fracture assessment
91301	Severe acute respiratory syndrome coronavirus 2 (SARS-CoV-2) (coronavirus disease [COVID-19]) vaccine, mRNA-LNP, spike protein, preservative free, 100 mcg/0.5 mL dosage, for intramuscular use (deprecated 2024)
91300	Severe acute respiratory syndrome coronavirus 2 (SARS-CoV-2) (coronavirus disease [COVID-19]) vaccine, mRNA-LNP, spike protein, preservative free, 30 mcg/0.3 mL dosage, diluent reconstituted, for intramuscular use (deprecated 2024)
0001A	Immunization administration by intramuscular injection of severe acute respiratory syndrome coronavirus 2 (SARS-CoV-2) (coronavirus disease [COVID-19]) vaccine, mRNA-LNP, spike protein, preservative free, 30 mcg/0.3 mL dosage, diluent reconstituted; first dose (deprecated 2024)
1011874	Immunology Procedures
A08	ANTIOBESITY PREPARATIONS, EXCL. DIET PRODUCTS
A08A	ANTIOBESITY PREPARATIONS, EXCL. DIET PRODUCTS
A10	DRUGS USED IN DIABETES
C01	CARDIAC THERAPY
C02	ANTIHYPERTENSIVES
C10	LIPID MODIFYING AGENTS
B01	ANTITHROMBOTIC AGENTS
J01	ANTIBACTERIALS FOR SYSTEMIC USE
J04	ANTIMYCOBACTERIALS
2683	colchicine

2468231	SARS-CoV-2 (COVID-19) vaccine, mRNA spike protein
R03	DRUGS FOR OBSTRUCTIVE AIRWAY DISEASES
CN000	CENTRAL NERVOUS SYSTEM MEDICATIONS
CN300	SEDATIVES/HYPONTICS
CN302	BENZODIAZEPINE DERIVATIVE SEDATIVES/HYPNOTICS
CN104	NON-STEROIDAL ANTI-INFLAMMATORY ANALGESICS
L02	ENDOCRINE THERAPY
L04	IMMUNOSUPPRESSANTS
59574-4	Body Mass Index Percentile
72166-2	Tobacco smoking status
9002	Cholesterol in LDL [Mass/volume] in Serum or Plasma
9001	Cholesterol in HDL [Mass/volume] in Serum or Plasma
9000	Cholesterol [Mass/volume] in Serum or Plasma
9004	Triglyceride [Mass/volume] in Serum, Plasma or Blood
9085	Blood Pressure, Systolic
9086	Blood Pressure, Diastolic
9014	Hemoglobin [Mass/volume] in Blood
4548-4	Hemoglobin A1c/Hemoglobin.total in Blood
9045	Albumin [Mass/volume] in Serum, Plasma or Blood
LG34557-5	Albumin/Creatinine [Mass ratio] in Urine
2003	Left Ventricular Ejection Fraction (LVEF) (%)
9039	Parathyrin.intact [Mass/volume] in Serum or Plasma
9040	Thyrotropin [Units/volume] in Serum, Plasma or Blood
1992-7	Calcitonin [Mass/volume] in Serum or Plasma
9035	Cortisol [Mass/volume] in Serum or Plasma
9022	Calcium [Mass/volume] in Serum, Plasma or Blood
2777-1	Phosphate [Mass/volume] in Serum or Plasma
8001	Glomerular filtration rate/1.73 sq M.predicted [Volume Rate/Area] in Serum, Plasma or Blood by Creatinine-based formula (MDRD)

TABLE S3.

Outcome Definitions

Table below outlines the definitions for each outcome and the analysis specifications. For outcome definitions consisting of more than one term, at least one term must match.

**Osteoporosis**			
	Diagnosis	UMLS:ICD10CM:M81.0	Age-related osteoporosis without current pathological fracture
	Diagnosis	UMLS:ICD10CM:M81	Osteoporosis without current pathological fracture
**T score in outcome /T ,Z score in exclusion criteria**			
	Laboratory	UMLS:LNC:38267-1	DXA Lumbar spine [T-score] Bone density (between -5.00 and -2.50 {T-score} (most recent occurrence))
	Laboratory	UMLS:LNC:80948-3	DXA Femur - left [T-score] Bone density (between -5.00 and -2.50 {Tscore} (most recent occurrence))
	Laboratory	UMLS:LNC:80947-5	DXA Femur - right [T-score] Bone density (between -5.00 and -2.50 {Tscore} (most recent occurrence))
	Laboratory	UMLS:LNC:85394-5	DXA Lumbar spine [Z-score] Bone density (between -5.00 and -2.0 0 {Zscore} (most recent occurrence))
	Laboratory	UMLS:LNC:80940-0	DXA Femur - left [Z-score] Bone density (between -5.00 and -2.00 {Zscore} (most recent occurrence))
	Laboratory	UMLS:LNC:80939-2	DXA Femur - right [Z-score] Bone density (between -5.00 and -2.00 {Zscore} (most recent occurrence))
	Laboratory	UMLS:LNC:38264-8	DXA Hip [T-score] Bone density (between -5.00 and -2.50 {T-score} (most recent occurrence))
	Laboratory	UMLS:LNC:80933-5	DXA Hip [Z-score] Bone density (between -5.00 and -2.00 {Zscore} (most recent occurrence))
	Laboratory	UMLS:LNC:80946-7	DXA Hip - left [T-score] Bone density (between -5.00 and -2.50 {Tscore} (most recent occurrence))

	Laboratory	UMLS:LNC:80938-4	DXA Hip - left [Z-score] Bone density (between -5.00 and -2.00 {Zscore} (most recent occurrence))
	Laboratory	UMLS:LNC:80945-9	DXA Hip - right [T-score] Bone density (between -5.00 and -2.50 {Tscore} (most recent occurrence))
	Laboratory	UMLS:LNC:80937-6	DXA Hip - right [Z-score] Bone density (between -5.00 and -2.00 {Zscore} (most recent occurrence))
	Diagnosis	UMLS:ICD10CM:R93.6	Abnormal findings on diagnostic imaging of limbs (between -5.00 and -2.00 {Zscore} (most recent occurrence))
**Fracture**			
	Diagnosis	UMLS:ICD10:s42	Fracture of shoulder and upper arm
	Diagnosis	UMLS:ICD10:s32	Fracture of lumbar spine and pelvis
	Diagnosis	UMLS:ICD10:s72	Fracture of femur
	Diagnosis	UMLS:ICD10:s82	Fracture of lower leg

Note: Codes were retrieved from the UMLS, ICD-10-CM, SNOMED CT, LOINC, and TriNetX databases. Laboratory criteria are based on the most recent available values unless otherwise specified. DXA scores correspond to standard diagnostic thresholds for osteoporosis (T-score ≤ -2.5).
